# Associations of Lifestyle Factors with Bone Mineral Density among Male University Students in Japan

**DOI:** 10.2188/jea.13.48

**Published:** 2007-11-30

**Authors:** Isuzu Egami, Kenji Wakai, Hirotada Kunitomo, Akiko Tamakoshi, Masahiko Ando, Toshiko Nakayama, Yoshiyuki Ohno

**Affiliations:** 1 Department of Food and Nutrition, Nagoya Bunri College.; 2 Department of Preventive Medicine/Biostatistics and Medical Decision Making, Nagoya University Graduate School of Medicine.; 3 School of Information Culture, Nagoya Bunri University.

**Keywords:** male youths, bone mineral density, nutrients, food groups, calcium, lifestyle

## Abstract

To investigate associations of lifestyle factors with bone mineral density among young men in Japan, we measured bone mineral density of the second metacarpal bone in 143 male university students, aged 18-22 years, by the computed X-ray densitometry. The subjects completed a lifestyle questionnaire including a quantitative food frequency questionnaire. Their mean±standard deviation of bone mineral density was 2.61±0.23 mmAl. Body mass index (Spearman’s *ρ*=0.232, p=0.006), daily walking time (*ρ*=0.186, p=0.028), and milk consumption at junior (*ρ*=0.250, p=0.003) and senior (*ρ*=0.195, p=0.020) high school were significantly correlated with the bone mineral density. For nutritional variables, the bone mineral density was positively correlated with energy-adjusted intakes of calcium (Pearson’s r=0.302, p=0.0002), potassium (r=0.265, p=0.001), saturated fatty acids (r=0.211, p=0.011), and magnesium (r=0.173, p=0.039), and with those of milk and dairy products (r=0.228, p=0.006) and fruits (r=0.205, p=0.014), while being negatively associated with energy-adjusted noodle consumption (r=-0.185, p=0.027). The positive correlation of milk consumption at junior high school with the bone mineral density was not materially altered by adjustment for the body mass index, calcium intake, and walking time. Single-life students had lower bone mineral density compared with those lived with families (p=0.044). Bone mineral density could be increased by modifying dietary habits in young men.

According to the Comprehensive Survey of the Living Conditions of People on Health and Welfare in Japan in 1998,^1^ becoming bedridden is mainly caused by cerebrovascular diseases (37.9%), senility (15.2%), and bone fractures and falls (12.4%) in the elderly aged 65 years or over. Disability by fracture will become more important in the aged population, since osteoporosis-related fractures have been increasing in both men and women.^2^ Bone mineral density (BMD) decreases with an advancing age, but several factors other than age also increase the risk of osteoporosis. Therefore, many studies have focused on such risk factors to find ways to prevent osteoporosis and its related fractures. The risk factors included estrogen deficiency,^3^^,^^4^ low dietary calcium intake,^5^^-^^8^ poor dietary habits,^5^^,^^9^ low physical activity,^5^^,^^10^ and genetic predisposition.^11^^,^^12^

Although osteoporosis is more common in elderly women, men also have an increasing risk of hip fracture with aging.^2^ Peak bone mass is estimated to be achieved during 16 to 30 years old.^13^^-^^16^ To attain a high peak bone mass in the young age may be an effective way to prevent osteoporosis.^17^ Identifying factors related to BMD will help young people achieve a greater peak bone mass.

Dietary factors have frequently been implicated in modifying bone health in young women,^9^^,^^17^^-^^19^ but their associations with BMD have rarely been examined in young Japanese men. We therefore related BMD to dietary factors in male university students, with special reference to associations between BMD and intakes of nutrients or food groups.

## METHODS

### Subjects

We recruited 163 subjects, aged from 18 to 22 years, from 280 male students who took the course for “Introduction to Health Education” in the Nagoya Bunri University. They participated in our study in January or December 2000. All these subjects provided informed consents.

Ten students taking calcium supplements were excluded. None of the participants took medication that interferes with calcium metabolism. We also omitted those with implausible energy intake estimated using a food frequency questionnaire (FFQ), that is, <1,100 kcal/day (n=6) or >4,500 kcal/day (n=4), leaving 143 participants (87.7% of those surveyed) eligible for the study (mean age ± standard deviation[SD]: 19.0 ± 0.7 years). Of these subjects, 110 (76.9%) lived with their families, while 33 (23.1%) led a single life. One third (n=46, 32.2%) smoked, and nine students (6.3%) had quitted smoking. Half of the participants (n=72, 50.3%) had an exercise habit at the time of survey.

### BMD measurement

BMD of the second metacarpal bone in the right hand was measured by the computed X-ray densitometry (CXD)^20^ using the BONALYZER (Teijin, Tokyo, Japan). The BMD was expressed in “mmAl”. The X-ray absoroption by one “mmAl” of BMD corresponds to that by an aluminum plate one millimeter thick. Information on anthropometric and medical factors was collected by a self-administered questionnaire.

### Dietary and other lifestyle factors

The subjects also filled out a questionnaire on dietary and other lifestyle factors. Its dietary part included a FFQ designed to assess diet during the past year and to estimate intakes of nutrients and food groups. The detailed design of FFQ used here has been described in a preceding report.^21^ The FFQ has good reproducibility and validity for estimating nutrient intakes^22^ and food group consumption.^21^ The correlation coefficient between the FFQ and 16-day dietary records for 18 nutrients ranged from 0.42 to 0.83(median: 0.61), and was 0.83 for calcium, 0.70 for potassium, 0.73 for saturated fatty acids (SFA), and 0.63 for magnesium. The corresponding coefficient for 16 food groups varied from 0.16 to 0.83 (median: 0.56), and was 0.83 for milk and dairy products, 0.80 for fruits, 0.64 for breads, and 0.42 for noodles.

The self-administered questionnaire also elicited other dietary and lifestyle factors: regularity of meals, preference for foods, current and previous intake frequencies of major caffeine sources (chocolate-containing foods, cola, and coffee), present and previous consumption of milk and small fishes, daily walking time, smoking habits, and current and previous exercise time. Almost every junior high school student is provided with milk at school lunch in Japan, then, from overall milk consumption in junior high school days, milk supplied at school lunch was excluded.

### Statistical analysis

Our study was conducted in two periods, namely, January (n=81) and December 2000 (n=62) and BMD level differed between the two groups of subjects, but overall associations of lifestyles with BMD were similar between the two groups (data not shown). Accordingly, we here presented the data as a whole.

Body mass index (BMI) was calculated as (body weight [kg])/(height [m])^2^. The two-sample *t* test was used to test mean differences between two groups. The associations of lifestyle factors with BMD were examined by use of the Spearman’s rank correlation coefficients (*ρ*), because the lifestyle variables could not easily be approximated to normal distribution.

Intakes of energy, nutrients, and food groups were estimated from the response to FFQ using a weighted food composition table. They were adjusted for energy intake by using the residuals from linear regression models.^23^ Energy and nutrient intakes and food group consumption were all natural-log transformed to improve their normality before the analyses. Pearson’s correlation coefficients were computed between BMD and energy-adjusted intakes of nutrients or food groups.

Multiple linear regression models^24^ were adopted for multivariate analyses. BMD was first regressed on energy-adjusted intakes of nutrients or food groups and on past milk consumption with adjustment for log_e_(BMI) and time of survey, that is, January or December 2000 (model 1). We further adjusted for energy-adjusted calcium intake (model 2). In the final model (model 3), the relationships between BMD and the dietary variables were examined, controlling for log_e_(BMI), energy-adjusted calcium intake, log_e_ (daily walking time), and time of survey.

We considered BMI, calcium intake, and daily walking time in the final model, because they were significantly associated with present BMD. BMI and daily walking time were also log_e_-transformed to improve normality. All *p* values were two-sided, and the statistical analyses were undertaken using the StatView^®^ version 5 for Windows^®^.^25^

## RESULTS

The BMI of study subjects ranged from 16.5 to 35.5 kg/m^2^ (mean ± SD: 20.9 ± 3.5 kg/m^2^), and their BMD from 2.07 to 3.38 mmAl with a mean ± SD of 2.61 ± 0.23 mmAl.

Table 1 presents Spearman’s rank correlation coefficients (*ρ*) between BMD and selected lifestyle factors. Those significantly correlated with BMD were BMI (*ρ*=0.232, p=0.006), daily walking time (*ρ*=0.186, p=0.028), and milk consumption during junior (*ρ*=0.250, p=0.003) and senior (*ρ*=0.195, p=0.020) high school days. Current and previous intake frequencies of major caffeine sources and those of small fishes as well were not associated with BMD (data not shown). Lower BMD was found in single-life students compared with those with families (mean ± SD: 2.54 ± 0.20 mmAl [n=33] vs. 2.63 ± 0.23 mmAl [n=110], p=0.044).

**Table 1.** Means and medians of selected lifestyle factors and Spearman’s rank correlation coefficients (*ρ*) between bone mineral density and the variables (n=143).

	Mean ± SD	Median	*ρ*	P
Age (years)	19.0 ± 0.7	19.0	-0.098	0.243
Body mass index (kg/m^2^)	20.9 ± 3.5	20.1	0.232	0.006
Smoking (no. of cigarettes/day)	4.7 ± 7.9	0.0	-0.121	0.148
Exercise during senior high school (min/day)	55.6 ± 72.3	0.0	0.031	0.708
Current physical exercise (min/day)	17.9 ± 44.1	0.0	0.080	0.340
Walking (min/day)	71.9 ± 93.7	40.0	0.186	0.028
Milk consumption at:				
Junior high school (glasses/day) ^a)^	1.23 ± 1.37	1.00	0.250	0.003
Senior high school (glasses/day)	1.06 ± 1.42	0.43	0.195	0.020

^a)^ Excluding milk provided at school lunch.

SD: standard deviation.

Table 2 summarizes mean daily intakes of nutrients and Pearson’s correlation coefficients (r) between energy-adjusted nutrient intakes and BMD. The BMD was positively correlated with intakes of calcium (r=0.302, p=0.0002), potassium (r=0.265, p=0.001), SFA (r=0.211, p=0.011), and magnesium (r=0.173, p=0.039). Fat (r=0.146, p=0.082) and vitamin C (r=0.162, p=0.054) intakes were weakly correlated with BMD.

**Table 2.** Mean daily intakes of nutrients and Pearson’s correlation coefficients (r) between energy-adjusted nutrient intakes and bone mineral density (n=143).

Nutrient	Mean	±	SD	r	p
Energy (kcal)	2184	±	713		
Protein (g)	65.8	±	23.3	0.122	0.148
Fat (g)	60.9	±	25.3	0.146	0.082
Carbohydrate (g)	320	±	103	-0.126	0.134
Calcium (mg)	447	±	286	0.302	0.0002
Iron (mg)	8.2	±	3.2	0.106	0.206
Potassium (mg)	2086	±	873	0.265	0.001
Vitamin A (IU)	1919	±	1046	0.117	0.165
Retinol (*μ*g)	360	±	228	0.058	0.492
Carotene (*μ*g)	1237	±	796	0.094	0.267
Vitamin C (mg)	63	±	48	0.162	0.054
Vitamin D (IU)	167	±	118	-0.029	0.727
SFA (g)	17.9	±	8.3	0.211	0.011
MUFA (g)	21.8	±	9.3	0.129	0.126
PUFA (g)	13.8	±	5.9	0.096	0.253
Cholesterol (mg)	260	±	164	0.114	0.174
Vitamin E (mg)	7.8	±	3.0	0.101	0.230
Dietary fiber (g)	10.6	±	4.4	0.060	0.474
Magnesium (mg)	242	±	83	0.173	0.039
Zinc (*μ*g)	8598	±	2823	0.129	0.124
Alcohol (ethanol, g)	6.1	±	24.6	0.041	0.625

SFA: saturated fatty acids, MUFA: monounsaturated fatty acids,

PUFA: polyunsaturated fatty acids.

SD: standard deviation.

Table 3 shows mean daily consumption of food groups and Pearson’s correlation coefficients between energy-adjusted intakes of food groups and BMD. Significant positive correlations were found between BMD and energy-adjusted consumption of milk and dairy products (r=0.228, p=0.006) and fruits (r=0.205, p=0.014). Energy-adjusted bread intake (r=0.156, p=0.062) was somewhat associated with BMD. A significant negative correlation was observed only for noodles (r=-0.185, p=0.027).

**Table 3.** Mean daily consumption of food groups and Pearson’s correlation coefficients (r) between energy-adjusted food group intakes and bone mineral density (n=143).

Food group (g/day)	Mean	±	SD	r	p
Rice	585	±	243	-0.128	0.128
Breads	27	±	32	0.156	0.062
Noodles	163	±	120	-0.185	0.027
Potatoes and starches	27	±	25	0.135	0.108
Confectioneries	34	±	28	-0.012	0.890
Fats and oils	17	±	9	0.045	0.593
Pulses	40	±	34	0.114	0.176
Fishes and shellfishes	35	±	24	-0.048	0.566
Meats	63	±	35	0.025	0.766
Eggs	27	±	28	0.057	0.498
Milk and dairy products	187	±	224	0.228	0.006
Vegetables	120	±	76	0.083	0.327
Green-yellow vegetables	48	±	51	0.114	0.176
Other vegetables	72	±	40	0.011	0.895
Fruits	102	±	115	0.205	0.014
Alcoholic beverages	72	±	182	-0.013	0.878

SD: standard deviation.

Table 4 summarizes the results from multiple linear regression analyses for associations of selected dietary factors with BMD. Dietary variables included in the analyses were those significantly or marginally correlated with BMD in the univariate analyses (Tables 1-3). In the model 1, BMD had positive correlations with daily intakes of calcium (*β*=0.126, p=0.001), potassium (*β*=0.213, p=0.005), SFA (*β*=0.165, p=0.010), magnesium (*β*=0.336, p=0.039), breads (*β*=0.025, p=0.069: marginally significant), milk and dairy products (*β*=0.039, p=0.007), and fruits (*β*=0.023, p=0.038), and milk consumption at junior (*β*=0.046, p=0.001) and senior (*β*=0.034, p=0.009) high school, independently of log_e_(BMI), and time of survey. A negative coefficient was found only for noodles (*β*=-0.031, p=0.085). Daily milk consumption at junior high school had similar correlations with BMD, even when energy-adjusted calcium intake was added to the model 1 (model 2: *β*=0.034, p=0.014). Additional adjustment for log_e_(daily walking time) did not materially alter this association (model 3: *β*=0.035, p=0.011). In contrast, no other dietary variables showed significant correlations with BMD, independently of energy-adjusted calcium intake. We further adjusted for number of cigarettes smoked per day, but the associations between BMD and dietary variables in the model 3 remained essentially unchanged (data not shown).

**Table 4.** Multiple linear regression analysis for associations between selected dietary factors and bone mineral density (mmAl, n=143).

Dietary factor (intake per day)	Model 1			Model 2			Model 3		
		
	*β* ^a^	SE	p	*β* ^b^	SE	p	*β* ^c^	SE	p
Fat (g)	0.133	0.077	0.088	-0.021	0.091	0.815	-0.009	0.091	0.924
Calcium (mg)	0.126	0.036	0.001						
Potassium (mg)	0.213	0.074	0.005	-0.010	0.134	0.941	-0.036	0.135	0.789
Vitamin C (mg)	0.039	0.029	0.171	-0.007	0.031	0.831	-0.015	0.031	0.641
SFA (g)	0.165	0.063	0.010	-0.019	0.100	0.854	0.011	0.101	0.911
Magnesium (mg)	0.336	0.162	0.039	-0.001	0.199	0.995	-0.004	0.198	0.986
Breads (g)	0.025	0.013	0.069	0.013	0.014	0.336	0.011	0.014	0.421
Noodles (g)	-0.031	0.018	0.085	-0.027	0.018	0.133	-0.026	0.017	0.145
Milk and dairy products (g)	0.039	0.014	0.007	-0.030	0.032	0.358	-0.027	0.032	0.405
Fruits (g)	0.023	0.011	0.038	0.008	0.012	0.507	0.006	0.012	0.617
Milk consumption at junior high school (glasses)^d^	0.046	0.013	0.001	0.034	0.014	0.014	0.035	0.014	0.011
Milk consumption at senior high school (glasses)	0.034	0.013	0.009	0.021	0.013	0.117	0.021	0.013	0.118

*β*: regression coefficient, SE: standard error, SFA: saturated fatty acids.

Intakes of nutrients and food groups were adjusted for energy intake. Energy and nutrient intakes and food group consumption were nat ural-log transfomed to improve their normality before the analyses.

^a^ Adjusted for log_e_ (BMI) and time of survey (January or December 2000).

^b^ Adjusted for log_e_ (BMI), energy-adjusted calcium intake, and time of survey.

^c^ Adjusted for log_e_ (BMI), energy-adjusted calcium intake, log_e_ (daily walking time), and time of survey.

^d^ Excluding milk provided at school lunch.

## DISCUSSION

We measured BMD by CXD method in the present study. This method can examine BMD in many subjects in a short time and has advantages of simple operation and high reproducibility.^26^ With regard to its validity, BMD measured by CXD well correlated with that by the dual energy X-ray absorptiometry (DXA),^27^ a standard method for BMD measurement. Matsumoto et al.^20^ reported that metacarpal BMD measured by CXD reasonably correlated with BMD in femoral neck (Pearson’s r=0.592), radius (r=0.837), and total body (r=0.664) determined by DXA.

The data of BMD measured by CXD method remain scarce in young men. The BMD in the present subjects (mean ± SD: 2.61 ± 0.23 mmAl), however, is comparable with BMD of men in similar age (20-24 years) measured using the same equipment as ours (2.72 ± 0.44 mmAl).^28^

We employed three regression models to examine the associations of dietary factors with BMD (Table 4). The model 1 may be used to see which food would be helpful to increase bone mass. The model 2 examined whether the associations of dietary variables were independent of calcium intake, one of the strongest nutritional determinants of BMD. Daily walking time was also considered in the model 3 because it was significantly related to BMD. The adjustment, however, did not materially alter the associations, and the model 3 may not be so informative.

Our cross-sectional study demonstrated a strong relationship between calcium intake and BMD in male university students. Calcium deficiency has been associated with lower bone mass in girls in their teens^14^^,^^17^ and in middle-aged^29^ or older^30^ adults. The associations in young men,^14^^,^^18^ however, have rarely been investigated in Japan. Our findings suggested that calcium intake during the young age would affect bone mass even in men. Young Japanese take much less calcium compared with Western people. In the United States, the mean daily calcium intake was 1,101 mg in men aged 19-30 years,^15^ while only 540 mg in males aged 20-29 years in Japan.^31^ Therefore, low calcium intake may be a risk factor of future osteoporosis and its related fractures, particularly in Japan. It may be worth noting, however, that osteoporosis-related fractures are not so common in Japan as in US^2^ albeit the low calcium intake in Japanese. The determinants of BMD other than calcium intake should be considered to explain this apparent paradox.

We also showed significant positive associations between BMD and intakes of potassium and magnesium, but they turned to be insignificant when considering calcium intake. This might probably be explained by strong correlations among intakes of calcium, potassium, and magnesium due in part to the overlap in dietary sources of these nutrients. We found a strong correlation of energy-adjusted calcium intake with that of potassium (Pearson’s r=0.842) or magnesium (r=0.613). In the present study, milk and dairy products were the largest dietary source of calcium (44.7%, estimated from the FFQ data), followed by pulses (11.3%), vegetables and fruits (8.7%), and fishes and shellfishes (4.4%). The second source, pulses, is rich in magnesium.^32^ The third source, vegetables and fruits, contains much potassium and magnesium.

SFA also had a positive correlation with BMD, but the correlation again disappeared after adjustment for calcium intake. This might largely be due to high calcium content in milk and dairy products that also contain considerable SFA. The Pearson correlation coefficient between energy-adjusted calcium and SFA intake was as high as 0.776 in our subjects. Intakes of bread and milk have a mutual association because of their well matching, which may explain the finding that the association of bread consumption with BMD was attenuated after controlling for calcium intake.

As for dietary history, our findings suggested that milk consumption in the growing period such as junior high school days increased BMD, independently of current calcium intake. Many studies^9^^,^^17^^,^^18^^,^^33^ have reported effects of milk drinking on BMD at young ages. Teegarden et al.^17^ found that higher milk consumption during adolescence was associated with a greater peak bone mass in young women, whereas current calcium intake could influence present BMD. In addition, milk intake at the younger age may consequently lead to more milk drinking in the later life.

The major sources of caffeine in children and teenagers are chocolate-containing foods, cola, coffee, and tea beverages.^34^ We attempted to assess the effect, if any, of caffeine consumption on BMD. Consumption of major caffeine sources during adolescence, however, was not correlated with BMD. Caffeine intake has been hypothesized to decrease BMD by increasing urinary calcium excretion in postmenopausal women.^35^ On the other hand, Lloyd et al.^34^ and Conlisk et al.^36^ reported no apparent relationship between caffeine intake and BMD in younger women. Our results in younger men were consistent with their findings.

Single-life students had lower BMD than those with families. They consumed less milk and dairy products (mean consumption per day: 74 g vs. 221 g, p=0.001 by *t* test for energy-adjusted values) and fruits (83 g vs. 108 g, p=0.023), compared with those lived with families. For nutrients, single-life students took less calcium (271 mg vs. 500 mg, p<0.0001), potassium (1,638 mg vs. 2,221 mg, p<0.0001), magnesium (211 mg vs. 252 mg, p<0.0001), vitamin C (51 mg vs. 67 mg, p=0.001), and SFA (14.4 g vs. 18.9 g, p=0.003). Intakes of these foods or nutrients were positively associated with an increased BMD. In addition, they showed shorter walking time per day (mean: 66 min vs. 74 min, p=0.008 by t test for natural-log transformed values) compared with those with family members. Our results, therefore, suggested that the poor nutrition and short walking time led to the lower BMD in single-life students. Ikai et al.^37^ reported that poor nutrition with an imbalanced diet was more frequently appeared in boarding female university students. They tended to skip a meal more frequently and to take fewer meals per week at their lodgings, which seemed to be due to an irregular daily life and lonely meals.

There appeared a positive correlation between BMD in the metacarpal bone and BMI. This may not be explained by mechanical loading imposed on bone. The bone mass, however, is also hormonally regulated. Recent studies^38^^,^^39^ have suggested that leptin stimulates osteoblastic differentiation and mineralization of bone matrix. Leptin is secreted by adipocytes, and its serum levels are strongly related to body fatness.^38^^,^^39^ Circulating leptin, therefore, may contribute to the association of BMI with BMD in the metacarpal bone.

The insulin-like growth factor-I (IGF-I) is another hormone that has extensively been studied in relation to bone resorption and formation.^40^ It also enhances osteoblastic differentiation and may play a role in maintaining bone mass. Physical activity may elevate serum levels of IGF-I.^41^ This might explain why BMD in the metacarpal bone positively correlated with walking time although walking does not directly give substantial mechanical stress to this bone.

One methodological limitation of this study is that our FFQ was validated only in a population largely different from that of this survey.^21^^,^^22^ Intakes of protein sources, vegetables, and several nutrients were much less in the present study than in the National Nutrition Survey,^31^ which may partly be ascribable to an underestimation by the FFQ. A validation study against dietary records or recalls is warranted in male university students. Another shortcoming of this FFQ is that we could not estimate intakes of phosphorus and vitamin K, which might have affected BMD.^19^^,^^42^^,^^43^ The physical activity questionnaire was not validated in this study. An error in the recall might have attenuated the association between past exercise and BMD, whereas daily walking time at the time of survey could more easily be reported.

Ferrari et al.^12^ suggested that environmental and dietary factors interact with polymorphisms of vitamin D receptor gene in affecting peak bone mineral mass in young men. Further investigations involving genetic markers will also be warranted.

In conclusion, the present study disclosed associations of dietary factors with BMD in male university students who have rarely been examined in Japan. Nutritional intakes or dietary habits were found to be important to increase BMD also in young men as in young women. Bone mineral density could be elevated by modifying dietary habits and increasing calcium intake at the young age. Longitudinal studies will certainly be necessary to verify the findings of our cross-sectional study, because such prospective investigations have rarely been conducted in young men in Japan, in particular.
